# Effect of Unit Cell Design and Volume Fraction of 3D-Printed Lattice Structures on Compressive Response and Orthopedics Screw Pullout Strength

**DOI:** 10.3390/ma18061349

**Published:** 2025-03-19

**Authors:** Boonyanuch Suksawang, Pisaisit Chaijareenont, Patcharawan Silthampitag

**Affiliations:** Department of Prosthodontics, Faculty of Dentistry, Chiang Mai University, Chiang Mai 50200, Thailand; boonyanuch_suk@cmu.ac.th (B.S.); pisaisit.c@cmu.ac.th (P.C.)

**Keywords:** additive manufacturing, stereolithography, lattice structure, screw pullout, triply periodic minimal surfaces

## Abstract

We aimed to evaluate the effects of unit cell design and the volume fraction of 3D-printed lattice structures with relative densities of 30% or 45% on compressive response and orthopedics screw pullout strength. All 3D lattice models were created using FLatt Pack software (version 3.31.0.0). The unit cell size of sheet-based triply periodic minimal surfaces (TPMSs)—Gyroid and Schwarz Diamond—was 5.08 mm, whereas that of skeletal TPMS—Skeletal Gyroid, Skeletal Schwarz Diamond, and Skeletal Schoen I-Wrapped Package—was scaled down to 3.175 and 2.54 mm. Two photopolymer resin types—Rigid 10k and Standard Grey—were used. In uniaxial compression tests, Rigid 10k resin lattices failed at relatively lower strains (<0.11), while Standard Grey lattices endured higher strains (>0.60) and experienced less softening effects, resulting in stress–strain curve plateauing followed by lattice densification. ANOVA revealed significant effects of design and volume fraction at *p* < 0.001 on compressive modulus, screw pullout strength, and screw withdrawal stiffness of the 3D-printed lattice. The pullout load from 3D-printed lattices (61.00–2839.42 N) was higher than that from open-cell polyurethane foam (<50 N) and lower than that of human bone of similar volume fraction (1134–2293 N). These findings demonstrate that 3D-printed lattices can be tailored to approximate different bone densities, enabling more realistic orthopedic and dental training models.

## 1. Introduction

Hands-on training programs focused on dental implant surgery have been integrated into dental education curricula to provide learners with a comprehensive understanding of anatomical structures and tactile feedback prior to engaging with patients. These preclinical training sessions enhance the confidence and skillset of these trainees, thereby contributing to improved implant survival rates and reduced complication risks during placements [1]. The models used in such training aim to replicate anatomical features and provide haptic feedback that mimics the sensation of working with real bone to optimize learning outcomes. Various polymeric models, made from materials such as polyurethane and synthetic foams, have been adopted in implant training programs. However, with high production costs, these models are commonly mass-produced in block form or generalized anatomic models, and their haptic feedback does not fully correspond to that of the actual bone tissue [2].

An effective approach to reducing costs while improving practical training for dental students is the internal production of replica models using 3D-printing technologies [3]. The use of additive manufacturing (AM) enables the construction of complex geometric shapes through a layer-by-layer process based on 3D computer-aided design (CAD) models. This technology provides a distinct advantage in producing models with intricate internal and external geometries [4]. Numerous benefits of incorporating 3D-printed models in dental education have been identified, such as enhancing the tactile comprehension of anatomical structures, practicing precise surgical pathways, and planning prosthetic devices, along with facilitating smoother intraoperative guidance and better communication between surgeons and patients [5].

The recent literature has focused on hands-on training with the use of 3D models and provided large-scale knowledge about the applicability in the medical and dental fields. Several studies have demonstrated the effectiveness of hands-on models created from cone beam computed tomography (CBCT) scans of real patients in oral surgery training, including procedures such as third-molar extractions, sinus lifts, and implant placements [6,7,8]. These studies also extend to orthognathic surgeries, providing valuable learning experiences for dental students [3,9]. Seifert et al. [10] conceived a comparative research study between 3D-printed patient-specific models and cadaveric animal models for oral and maxillofacial surgery training. It was concluded that 3D-printed models produced using cost-effective technology provided realistic anatomical accuracy and operationally simulated ones. Whereas the cadaveric models had a higher rating regarding haptic feedback in soft tissue manipulation, the 3D-printed models did much better in terms of ratings on anatomical precision, freedom of movement, and cost-effectiveness and can thus be considered as an alternative for surgical education. In this respect, studies such as those by Wang et al. [2] and Shujaat et al. [11]—in developing 3D-printed dental models for implant and screw insertion—established material and printing technology as playing a critical role in determining model performances. For instance, Wang et al. [2] reported that, regarding drilling simulation and implant placement, the haptic feedback of models printed by material jetting technology with acrylic-based resin was superior to that of models generated using other techniques. The results have shown positive prospects for integrating such models in dental implant simulation exercises. However, none of the models tested had a tactile feel that was exactly like real bone, and thus materials science and resolution need to evolve further. Moreover, Shujaat et al. [11] mentioned that the properties of the base material substantially influence the performance of the hands-on model; for example, gypsum-like material offered the highest rating for performing osteotomy cuts, while a low rating was observed for drilling holes, screw insertion, and removal. Conversely, nylon-like material was the least favorable for performing surgery. Collectively, these studies illustrate the growing potential of 3D-printed hands-on models in dental and medical education. They offer practical solutions to challenges such as cost, accessibility, and anatomical accuracy while highlighting areas for improvement in material selection and haptic feedback optimization to further bridge the gap between synthetic and real bone models.

Nonetheless, a key limitation is that most existing studies primarily assess qualitative features of these models, without a detailed examination of their mechanical properties, which could influence how users perceive the tactile feedback. Additionally, while CBCT offers valuable insights into patient anatomy, it does not capture the intricate details of internal trabecular bone structures as effectively as microcomputed tomography (CT) [12]. Although AM allows for the creation of synthetic bone models, the accuracy of such models is constrained by the resolution capabilities of the 3D-printing equipment, particularly at the level of trabecular bone architecture [13]. Some studies attempted to print trabecular bone replicas with a 3D model exported directly from the patient’s micro-CT [13,14]. The 3D model was further processed using dilation methods to thicken the feature size and was subsequently scaled up to various folds before the printing process. The two cited studies used the same micro-CT source file but with different printing technologies and materials.

The materials science field has drawn inspiration from nature, as evidenced by the development of cellular materials [15]. Studies comparing natural and industrial materials [16,17] have shown that while the mechanical properties of these materials are similar, natural materials tend to be lighter in weight. From a materials science perspective, cortical bone corresponds to a dense solid, whereas trabecular bone represents a cellular form of the same material, which is a nanocomposite of hydroxyapatite and collagen [18,19].

Recent improvements in AM technology have made it possible to create complex metamaterials like lattice structures with a regularly recurring minute unit, which can be implemented as space-filling structures to improve production qualities in various industries [20]. The unit cell represents the fundamental building block that forms and defines the overall structure. The mechanical properties of these structures can be predicted based on the geometries of their unit cells, along with factors such as relative density, size, strut dimensions, and overall arrangement [4]. Various methodologies have been introduced for creating scaffolds with lattice structures, often beginning with the development of an elementary unit cell in CAD programs, which is subsequently organized within a 3D space to form a complete shape.

Lattice structures encompass various configurations, including 3D strut-based models like Octet truss and Kelvin lattices, as well as honeycomb structures and 3D sheet or network-based lattices, such as those derived from triply periodic minimal surfaces (TPMSs). TPMSs are characterized by infinite, periodic lattice structures extending in three principal directions without self-intersection. Minimal surfaces are distinguished by having zero mean curvature across all points. These surfaces, which form TPMSs, exhibit periodicity in three independent directions, dividing space into two interconnected labyrinth-like domains with continuous joints and smooth curvatures formed through basic trigonometric functions [21]. The appeal of TPMS-based lattices lies in their remarkable features, including the ability to control pore size, strut size, curvature, and volume fraction and the capability of functional grading. These features represent them as potentially attractive options regarding a wide range of applications, including the construction, electrical, acoustic, transportation, chemical, optical, and energy industries. However, these structures are particularly promising for bone tissue engineering due to their good fluid dynamic characteristics, high energy absorption, and favorable surface-to-volume ratio [22].

Lattice structures have two main types of mode of deformation: stretching-dominated and bending-dominated. For a given relative density, cellular structures with stretching-dominated behavior have greater stiffness and strength than those characterized by bending-dominated deformation. However, structures that are bending-dominated typically demonstrate greater energy absorption capacity compared to their stretching-dominated counterparts [23]. Bending-dominated structures, which are structurally weaker than their stretching-dominated counterparts with the same density, exhibit a more gradual failure under compression forces. This behavior reduces sudden drops in load capacity during buckling and mirrors the irregular structural patterns observed in human bone [24].

An important finding is that sheet-based TPMS lattices display various deformation modes, combining elements of both stretching and bending, but predominantly show stretching-dominated behavior. By contrast, all examined ligament-based TPMS lattices exhibited a bending-dominated deformation mode, which suggests that they are highly effective for applications requiring energy absorption [25]. Solid-network TPMS lattices can be viewed as open-cell periodic foams, whereas sheet-network TPMS lattices are akin to partially closed-cell structures where interconnected pores span a 3D space. These interconnected networks contrast with fully closed-cell lattices that feature isolated pores [21]. Although solid-based TPMS generally possess lower mechanical strength compared to sheet-based configurations, research on how these variations affect screw pullout strength remains limited.

Notable advancements in the use of biocompatible lattice structures for orthopedic implants have been documented. By manipulating the porosity and stiffness of these structures, they can serve as effective scaffolds for promoting the integration of bone and tissue while also helping to alleviate the issue of stress transfer commonly associated with solid implants. These porous designs play a crucial role in bone tissue engineering and are widely employed in orthopedic clinics due to their low Young’s modulus, high compressive strength, and ample space for cellular growth [26]. Despite their extensive application in synthetic bone engineering, studies focusing on the incorporation of lattice structures into practical hands-on models remain scarce. Hence, the current study aimed to assess how varying design parameters influence the mechanical behavior of lattice structures produced using two distinct types of photopolymer materials.

## 2. Materials and Methods

### 2.1. Lattice Structure Design

The average trabecular thickness of the jawbone determined from micro-CT scans ranges from 0.22 mm to 0.57 mm depending on the anatomical site and imaging technique [12,27,28,29,30]. The trabecular thickness of dentate alveolar bone is larger than that of atrophic alveolar bone [31,32].

The microstructure of trabecular bone is usually shown as a collection of plate-like to rod-like microarchitecture. Findings from both computational models and experimental observations suggest that trabecular plates are more influential than rods in maintaining the mechanical stability of human trabecular bone [33].

Bone density (i.e., bone tissue volume to total volume [BV/TV]) also varies among anatomic regions of the jawbone and depends on multiple factors both local (atrophy, edentulous) and systemic (age, sex, osteoporosis). In the systematic review by Monje et al., the BV/TV value ranged from 10.8% to 62.2% with a mean value of 36.5% [32].

**Figure 1:**
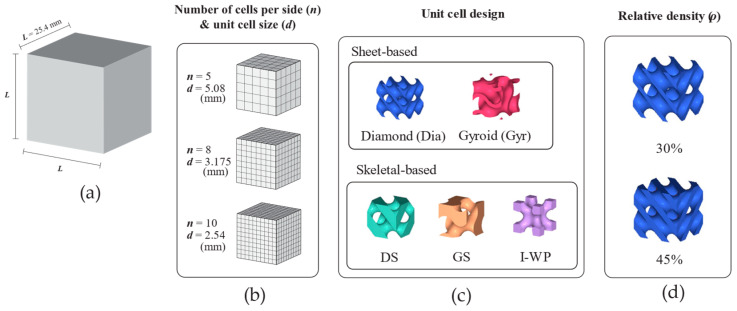
Design schematic of lattice structures. (**a**) Lattice dimension. (**b**) Number of unit cells per side of lattice structure and unit cell size. (**c**) Unit cell design. (**d**) Relative density. Abbreviations: Dia—Schwarz Diamond; DS—Skeletal Schwarz Diamond; GS—Skeletal Gyroid; Gyr—Gyroid; I-WP—Skeletal Schoen I-Wrapped Package.

The design groups in this study started with two sheet-based TPMSs with unit cell counts of *n* = 5 per side, which resulted in an average part thickness close to that of trabecular bone. Due to limitations in time and computational resources, the design groups were limited, since further decreasing the volume fraction or increasing the unit cell number would increase both CAD file size and processing time. Additionally, three skeletal-based TPMSs were included in the design groups. In the modeling phase, these structures were initially designed as cubic unit cells before being multiplied in 3D space to occupy the assigned dimensions. The lattice specimen dimensions and design parameters are shown in Figure 1.

In the case of lattice structures, two relative densities were considered, namely, 30% and 45%. All 3D lattice models were generated using FLatt Pack software (version 3.31.0.0) [34]. Regarding sheet-based TPMS, the current study investigated two types (Gyroid and Schwarz Diamond), whereas among skeletal TPMSs, the study considered three types (Skeletal Gyroid, Skeletal Schwarz Diamond, and Skeletal Schoen I-Wrapped Package; Figure 1c). The sheet-based lattices were only modeled using 5-unit cell tessellates in each direction. For the skeletal-based lattice design, the unit cell size was also scaled down to 8- and 10-unit cells per side to study the effect of unit cell size.

The lattice design was then combined into 22 groups for each material. The designed lattices were subsequently processed using the thickness analysis module in SolidWorks software (version 2022 SP2.1, Dassault Systèmes, Vélizy-Villacoublay, France) to measure the average feature size (Table 1).

### 2.2. Specimen Fabrication

The CAD files were saved in stereolithography (STL) format and subsequently processed using PreForm software (version 3.28.0; Formlabs, Somerville, MA, USA) to set up the printing tasks. The software automatically configured the required support for printing. The samples were then printed using the Form 3L printer (Formlabs), which utilizes low-force stereolithography (SLA) technology.

Two types of photopolymer resin (Formlabs), “Rigid 10k resin” and “Standard Grey resin”, with different material properties were used in this study (Table 2). Four specimens for each material were printed for one lattice design. In the PreForm software, layer height was set to 100 µm with auto-generated support structure. While technologies that rely on chemical reaction-based solidification or bonding, like PolyJet and stereolithography (SLA), also exhibit some mechanical anisotropy when the build orientation changes, they are less susceptible to such orientation variations compared to methods like material extrusion and powder bed fusion [35,36,37]. In the current study, all lattice structures were printed in the same orientation (0° building angle) to reduce the impact of anisotropy on mechanical properties, ensuring a fair comparison among the tested lattice structure designs. After the printing process, all 3D-printed specimens were submerged in isopropyl alcohol for 10 min to eliminate residual liquid resin. Following this step, the samples underwent UV curing for 120 min at 60 °C. Subsequently, the support material used to hold overhanging parts was detached using flush cutters. The accuracy of the 3D printer was verified by determining the weight of the samples using a digital scale with a precision of 0.0001 g (Mettler Toledo, Columbus, OH, USA). Each weight was then compared with that calculated from the mass of the CAD model. The differences in weight were within 3% of the predicted values based on the CAD models, which were calculated from the material density of the photopolymer resins.

### 2.3. Mechanical Tests

**Figure 2:**
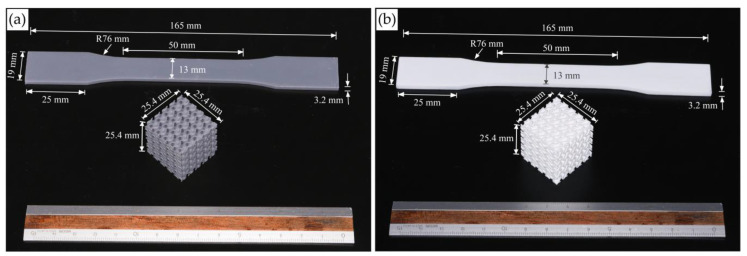
Images of mechanical test specimens printed with Standard Grey (**a**) and Rigid 10k (**b**) resin.

The mechanical properties of the photopolymer materials were evaluated using tensile tests based on the ASTM D638-14. Five specimens printed with the same setting as that of lattice specimens were tested for each type of resin. Figure 2 shows test specimens printed from both photopolymer resins. For the tensile tests, the specimens were tested using the Instron 5566 universal testing machine (Instron, Norwood, MA, USA) with a 10 kN load capacity with a crosshead speed of 5 mm/min.

Uniaxial quasi-static compression tests were conducted to evaluate the compressive and deformation responses of lattice blocks. Four samples from each lattice structure design were tested following the ASTM D1621-00 standard. The specimens were placed between two steel plates of the testing machine, with the lower plate fixed while the upper plate moved at a consistent speed of 2.5 mm/min. The tests were halted once 60% plastic strain was achieved or when the structure became dense or failed. Video recordings captured during the tests helped identify any failure modes in the samples. Force–displacement (F-d) curves were derived from these tests and later transformed into nominal stress-strain curves. The nominal stress (*σₙ*) was calculated by dividing the applied force by the initial cross-sectional area of the lattice, and the nominal strain (*εₙ*) was determined as the ratio of the change in height to the initial height of the specimen. Four samples from each design were assessed, with all measured data recorded and the corresponding engineering stress–strain curves generated.

The mechanical properties of lattice structures are influenced by their relative density, as described by the Gibson–Ashby scaling power law presented in Equation (2). The relative density ($\rho^{*})$ is defined in Equation (1) as the ratio of the apparent density ($\rho^{L})$ of a lattice structure to the material density $\left(\rho^{S}\right)$. Regardless of the topology of the lattice structure, it is observed that the strength of the lattice structure diminishes as the relative density $\rho^{*}$ decreases.

(1) $$\rho^{*}=\frac{\rho^{L}}{\rho^{S}}$$

The Gibson and Ashby model establishes a correlation between the relative density of a cellular structure and its corresponding relative strength or modulus.

(2) $$\frac{E^{L}}{E^{S}}=C{\left(\rho^{*}\right)}^{n}$$

The parameters $E^{L}\;and\;\rho^{L}$ represent the modulus and density of the lattice structure, whereas $E^{s}\;and\;\rho^{s}$ refer to those of the base material. The variable *C* is a geometric factor influenced by the lattice topology, loading direction, manufacturing flaws, and boundary conditions. The value of *n* defines the various deformation modes (such as stretching, bending, or a mixed mode) of cellular structures [24]. A value of *n* ≈ 1 suggests a stretching-dominated mode, whereas values of *n* ≥ 2 imply higher stiffness, and *n* > 1.5 indicates a bending-dominated mode. Other values of *n* point to a mixed deformation mode.

The parameters *C* and *n* can be determined through experimental methods by fabricating and testing lattice samples with different volume fractions, followed by applying appropriate curve fitting techniques to the mechanical data. Alternative approaches, such as finite element analysis and the development of analytical models that focus on structural failure mechanisms, can also be used [34].

Using FLatt Pack software, the elastic modulus of various cell types across a range of volume fractions was computed via the finite element homogenization method.

**Figure 3:**
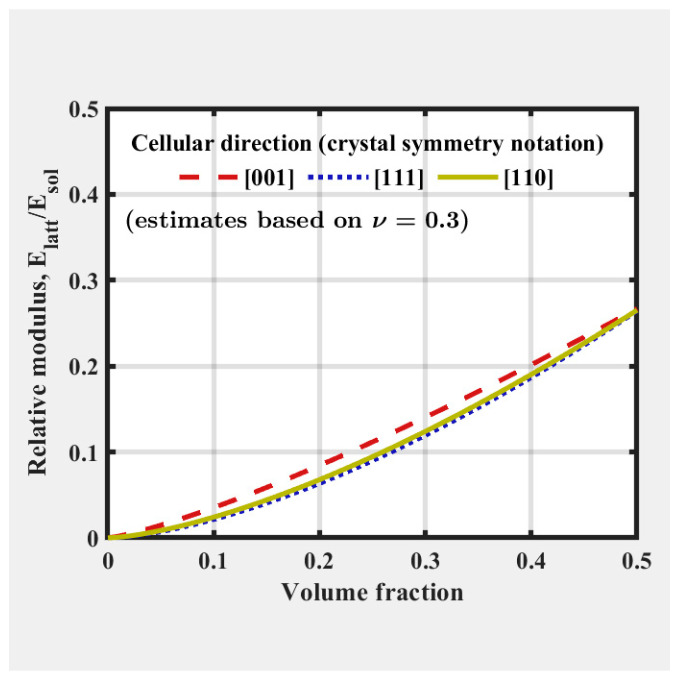
FLatt Pack software user interface which shows the relative modulus-relative density of the selected unit cell type [34].

As the moduli of regularly ordered lattices are typically anisotropic, the software provides relative modulus-relative density curves, shown in Figure 3, along specific high symmetry directions such as [001], [111], and [110], which follow crystallographic notations for cubic lattice structures.

### 2.4. Screw Pullout Test

**Figure 4:**
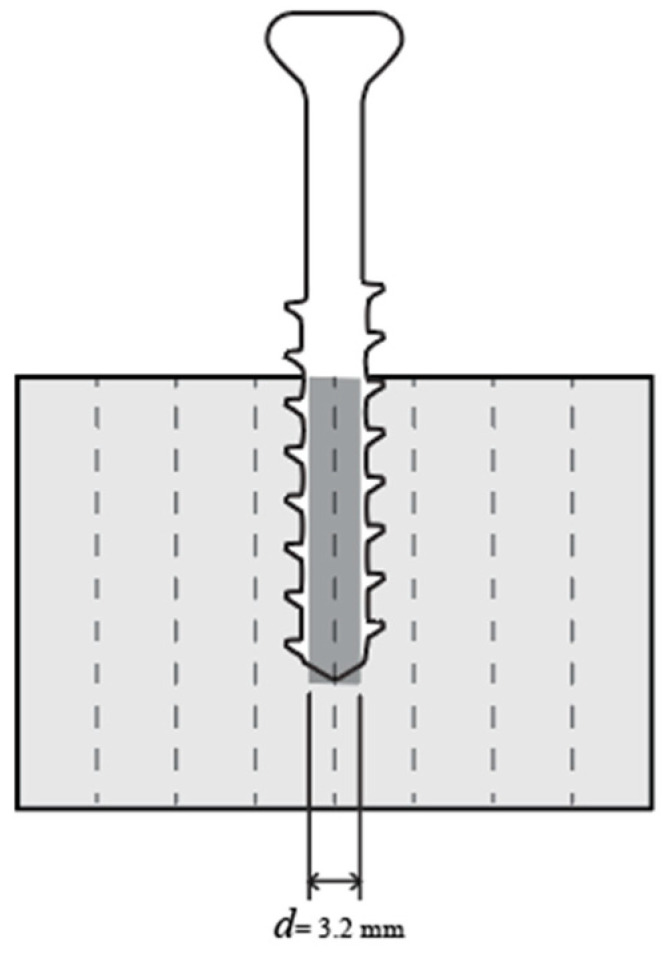
Illustration of the pullout specimen drilling position in cross-sectional view.

**Figure 5:**
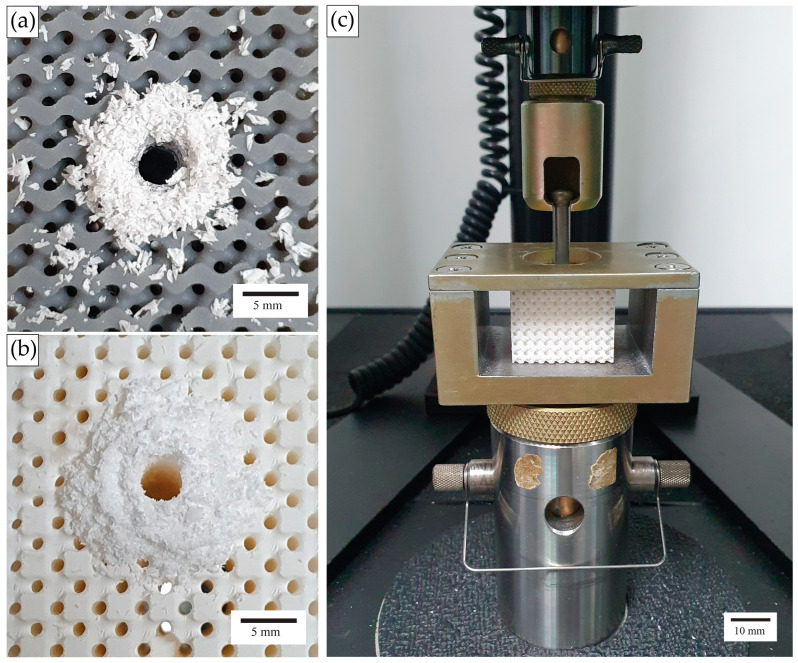
(**a**,**b**) Images of lattice specimens after predrilling (Standard Grey specimen and Rigid 10k specimen, respectively) and (**c**) a screw pullout experimental setup.

The screw pullout tests of printed lattice specimens were performed in accordance with the ASTM F543 standard. Each lattice specimen was predrilled at the intersection of adjacent unit cell corners with a hole diameter of 3.2 mm and a depth of 20 mm, as illustrated in Figure 4. A Titanium HB 6.5 orthopedic cancellous screw (Jiangsu IDEAL Medical Science & Technology, Zhangjiagang, China) was inserted manually into each predrilled hole. The screws were then extracted from the lattice structures using a universal testing machine (Figure 5c), ensuring a consistent extraction speed of 1 mm/min. There are four replicates of each design tested. Among those, the skeletal TPMS of a 5.08 mm cell size was excluded when studying Rigid 10k lattices for this configuration due to lattice damage during screw insertion.

### 2.5. Statistical Analysis

The statistical analysis was carried out using SPSS Statistics software (version 25, IBM, Armonk, NY, USA). A two-way analysis of variance (ANOVA) was conducted to examine the impact of volume fraction and unit cell design on compressive modulus. Additional statistical tests were performed to assess the effect of these factors on screw pullout strength and screw withdrawal stiffness. To determine the differences between groups, Tukey’s honestly significant difference post hoc test was applied. Statistical significance was set at *p* < 0.05. The total sample size was *N* = 88 for each material in both the compression test and the screw pullout test.

## 3. Results

### 3.1. Quasi-Static Compression Test

**Figure 6:**
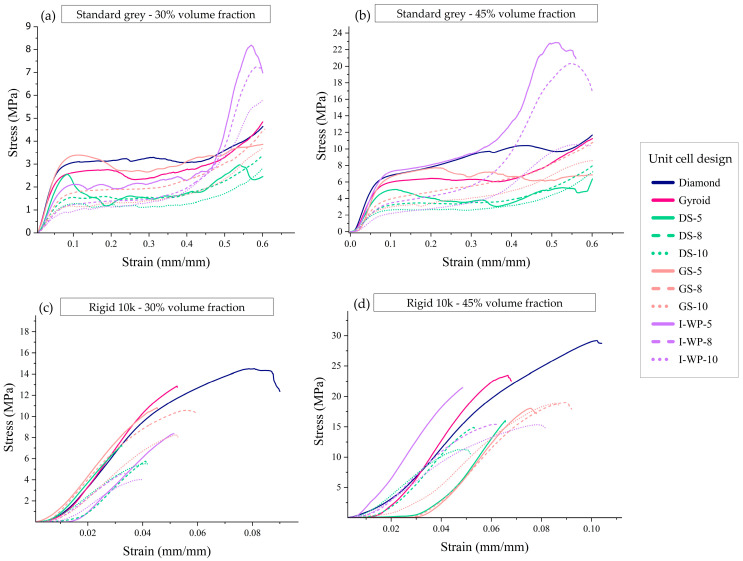
Engineering stress–strain curves from uniaxial compression of Standard Grey lattices with 30% volume fraction (**a**), Standard Grey lattices with 45% volume fraction (**b**), Rigid 10k lattices with 30% volume fraction (**c**), and Rigid 10k lattices with 45% volume fraction (**d**). Abbreviations: DS—Skeletal-Schwarz Diamond; GS—Skeletal Gyroid; I-WP—Skeletal Schoen I-Wrapped Package.

The typical responses of each lattice design are illustrated in Figure 6, showing the relationship between macroscopic nominal stress (*σ*), calculated as force divided by undeformed area, and average strain, which is derived from shortening (*δ*) divided by original height (*h*^0^), up to 60% strain or structural failure.

Rigid 10k resin lattices endured relatively lower strain (<0.11) than Standard Grey lattices (>0.60). The plotted curves demonstrate that the responses of the Rigid 10k lattices were similar and almost perfectly linear elastic until they reached a critical stress. In contrast, the Standard Grey specimens experienced fewer softening effects, resulting in the stress–strain curve leveling off into a plateau after the yield point (Figure 6).

The compressive stress–strain curves for Standard Grey lattice structures displayed three distinct regions. The first is the initial linear elastic region, which exists between zero stress and the yield stress. The second is the plateau region, spanning from compressive yield strength to densification strain, where the unit cell structures collapse and buckle under increasing applied force. The third region is the densification area, which occurs after densification strain, during which the structures contact each other, thereby increasing resistance to further deformation.

According to Yang et al. [38], the number of cells is the most influential factor for enhancing the elastic modulus, followed by wall thickness. The third and fourth most important factors are the bulk size and iso-value, respectively. A similar trend was observed for compressive strength, with increasing the number of cells having the greatest impact, followed by wall thickness, bulk size, and an iso-value near 0.5.

Reducing the feature size at lower volume fractions leads to structural instability under compressive loads, which ultimately results in global failure. This phenomenon could account for the multiple failure locations that evolve into multiple failure planes in designs with small cell sizes. On the other hand, designs with larger cell sizes and thicker feature sizes provide greater structural rigidity, which restricts failures to localized areas.

The findings from the study demonstrate that a unit cell design within a fixed relative density lattice model had a notable impact on the compressive strength and energy absorption characteristics of the lattice. Furthermore, having a specific number of cells within a defined lattice block volume improved the structural rigidity and enhanced the energy absorption performance.

**Figure 7:**
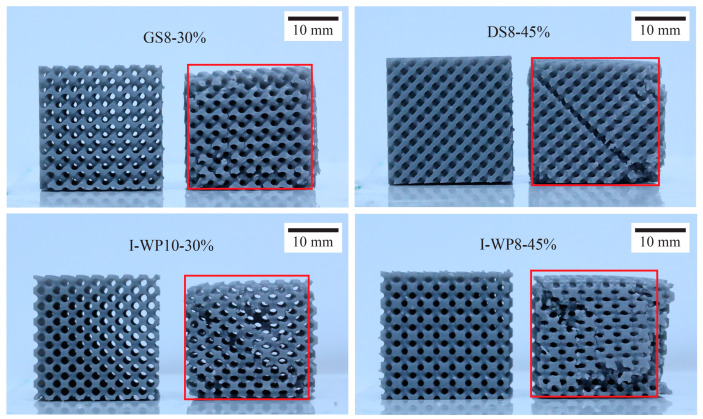
Standard Grey specimens up to 20 min after termination of compression test next to untested specimens. Abbreviations: GS—Skeletal Gyroid; DS—Skeletal Schwarz Diamond; I-WP—Skeletal Schoen I-Wrapped Package.

**Figure 8:**
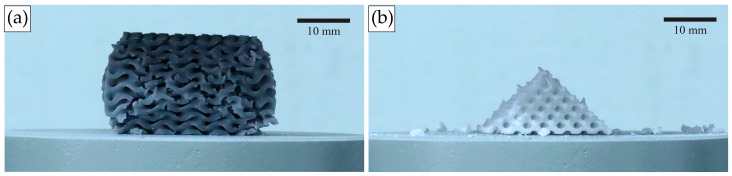
Specimens undergoing uniaxial compression test. (**a**) Standard Grey lattices, after becoming densified at 60% compressive strain, rebound after test termination. (**b**) In Rigid 10k lattices, cracks propagate through the structure leading to catastrophic failure.

Structures printed from Standard Grey material could be compressed to up to 60% strain before entering a densification state. After unloading, Standard Grey specimens gradually returned to 85–90% of their original height (Figure 7). However, structures printed using Rigid 10k material underwent fracture at relatively low strain levels owing to the brittleness of the parent material, and the resulting fragmented pieces could not be further compressed (Figure 8).

**Figure 9:**
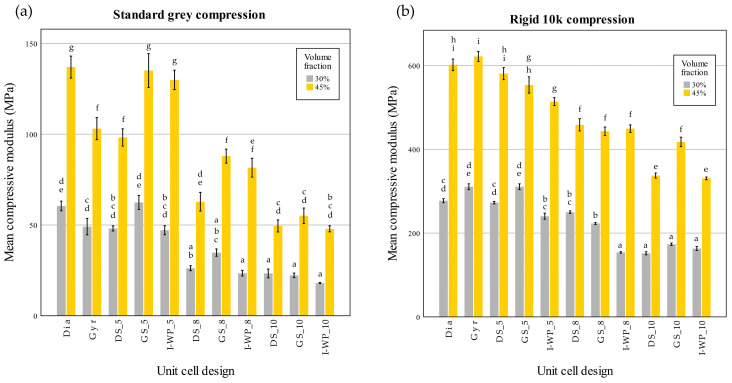
Compressive modulus values of Standard Grey (**a**) and Rigid 10k (**b**) lattices. Similar statistical letters on each bar indicate non-significant difference among the lattice design of each material in Tukey’s HSD post hoc test. Abbreviations: Gyr—Gyroid; Dia—Schwarz Diamond; GS—Skeletal Gyroid; DS—Skeletal Schwarz Diamond; I-WP—Skeletal Schoen I-Wrapped Package.

The compressive modulus and compressive strength values of each design were plotted to observe the trends among the different design groups (Figure 9).

Relative density is another factor that greatly impacts a structure’s compressive modulus and compressive strength. Notably, certain designs with a density of 45% may have lower strength than structures with a density of 30%. Regarding cost-effectiveness, manufacturers may consider printing structures with a density of 30%, as this would consume less resin during production. This approach could potentially reduce production costs while maintaining desired properties.

Among the lattice types examined in this study, diamond lattices demonstrated superior performance regarding toughness and ductility.

A two-way ANOVA was conducted to evaluate the influence of volume fraction and unit cell design on compressive modulus. The results from the Standard Grey lattices indicated a significant interaction between these factors (F(10,66) = 11.011, *p* < 0.001). Further simple main effects analysis confirmed that both the volume fraction and unit cell design had significant impacts on compressive modulus (*p* < 0.001 for both).

The findings from the Rigid 10k lattice tests demonstrated a significant interaction between the volume fraction and unit cell design (F(10,66) = 17.417, *p* < 0.001). Subsequent simple main effects analysis confirmed that both factors significantly influenced the compressive modulus (*p* < 0.001 for both). These analyses were then extended with Tukey’s honestly significant difference post hoc test to assess pairwise comparisons at *p* < 0.05.

From the post hoc test of the Standard Grey lattices, for design with 30% volume fraction, no significant difference was found between each unit cell design of the same unit cell size. For the Standard Grey lattices with 45% volume fraction the differences among shapes became less pronounced as the unit cell size decreased, with no significant difference between the 10-cell groups (Figure 9a). For both materials, the lattice design of 30% volume fraction demonstrated compressive modulus values with no significant difference from the design of 45% volume fraction.

Furthermore, Pearson’s correlation coefficient revealed a strong relationship between lattice density and compressive properties (Standard Grey r = 0.703; Rigid 10k r =0.843), whereas a moderate correlation was observed between the average part thickness and compressive properties (Standard Grey r = 0.561; Rigid 10k r = 0.417), and all relationships were statistically significant (*p* < 0.01).

### 3.2. Screw Pullout Test

In this study, three designs of Rigid 10k material (all skeletal TPMSs of 45% volume fraction at 5.08 mm cell size) could not undergo the screw pullout test, since most surrounding struts failed during screw insertion, and no valid data were obtained. Data from the screw pullout test are shown in Table 3 and Table 4. The screw pullout strength values ranged from 174.38 to 1466.50 N for the Standard Grey lattices and 61.00 to 2839.42 N for the Rigid 10k lattices. Interestingly, the lattices that demonstrated the highest and lowest screw pullout valued were of a different design for each material.

**Figure 10:**
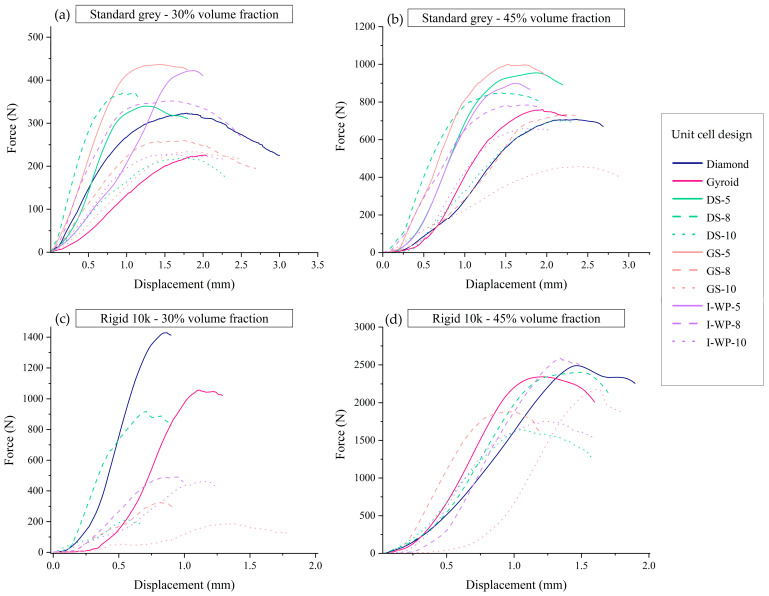
Force–displacement curves from screw pullout tests of Standard Grey lattices with 30% volume fraction (**a**), Standard Grey lattices with 45% volume fraction (**b**), Rigid 10k lattices with 30% volume fraction (**c**), and Rigid 10k lattices with 45% volume fraction (**d**). Abbreviations: GS—Skeletal Gyroid; DS—Skeletal Schwarz Diamond; I-WP—Skeletal Schoen I-Wrapped Package.

Figure 10 illustrates the force–displacement responses obtained during screw pullout tests for four different groups of lattice specimens. Each panel a–d corresponds to a specific combination of base material (Standard Grey vs. Rigid 10k) and volume fraction (30% vs. 45%). In all graphs, the *x* axis denotes the displacement of the screw during its extraction from the lattice specimen, whereas the *y* axis represents the measured pullout force. The peak of each curve indicates the maximum load at which the screw–lattice interface fails.

Figure 10a (Standard Grey, 30% volume fraction): In this graph, the screw pullout forces generally reached moderate peak values, in the range of a few hundred newtons, before experiencing a sharp drop that reflects localized strut failures around the screw threads. Designs featuring thicker struts or more robust topologies (e.g., sheet-based TPMSs) tend to exhibit higher peak forces, whereas skeletal-based TPMSs with smaller cell sizes display lower but more gradual load–displacement profiles. Notably, the displacement at failure can be relatively large, indicating a certain degree of ductility and progressive damage prior to the abrupt load reduction.

Figure 10b (Standard Grey, 45% volume fraction): When the volume fraction increased to 45%, the maximum pullout load rose substantially. Most curves in this panel exhibited pronounced peaks exceeding those observed in Figure 10a, suggesting that the higher material content and thicker features enhance the screw’s anchorage. In addition, some curves show a steeper initial slope indicative of greater initial stiffness. After reaching the peak load however, the force dropped sharply as the lattice material fractured around the screw, and the load-bearing capacity diminished.

Figure 10c (Rigid 10k, 30% volume fraction): These lattice specimens demonstrate noticeably higher peak forces than their Standard Grey counterparts at the same density. This reflects the intrinsically higher stiffness and strength of the Rigid 10k resin. However, the initial slope of most curves is quite steep, underscoring an elevated resistance to deformation despite the still relatively low lattice density. Beyond the peak force, the curves typically drop off abruptly, indicating a brittle failure mode where fractured struts quickly lose their load-carrying capacity.

Figure 10d (Rigid 10k, 45% volume fraction): At 45% volume fraction, the Rigid 10k specimens showed the highest overall pullout strengths among all tested groups, frequently exceeding 2000 N. The enhanced material content, combined with the inherently stiff resin, leads to both large peak forces and steep slopes in the elastic region. However, as with the other groups, the load dropped abruptly post-peak, emphasizing the brittle, sudden nature of strut failure. It is also noteworthy that certain skeletal TPMS designs at this density and cell size were excluded from testing due to premature strut breakage during screw insertion, highlighting the interplay between unit cell geometry, lattice density, and brittle resin properties.

**Figure 11:**
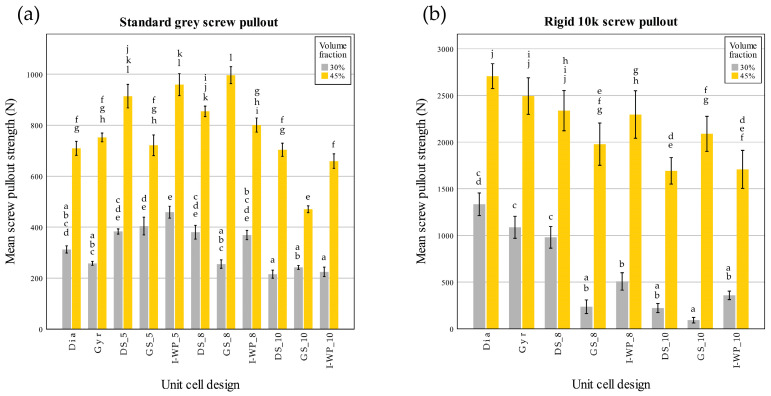
Screw pullout force (N) of Standard Grey (**a**) and Rigid 10k (**b**) lattices. Similar statistical letters on each bar indicate non-significant difference among the lattice design of each material in Tukey’s HSD post hoc test. Abbreviations: Gyr—Gyroid; Dia—Schwarz Diamond; GS—Skeletal Gyroid; DS—Skeletal Schwarz Diamond; I-WP—Skeletal Schoen I-Wrapped Package.

**Figure 12:**
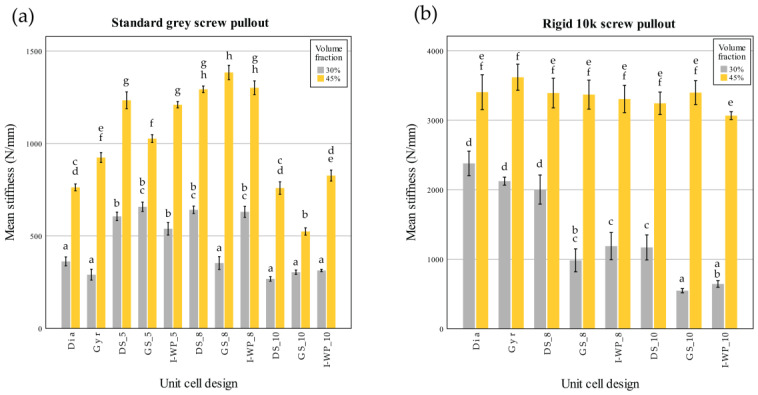
Screw withdrawal stiffness (N/mm) of Standard Grey (**a**) and Rigid 10k (**b**) lattices. Similar statistical letters on each bar indicate non-significant difference among the lattice design of each material in Tukey’s HSD post hoc test. Abbreviations: Gyr—Gyroid; Dia—Schwarz Diamond; GS—Skeletal Gyroid; DS—Skeletal Schwarz Diamond; I-WP—Skeletal Schoen I-Wrapped Package.

These findings in Figure 10 correspond closely to the data shown in Figure 11 and Figure 12, which summarize the screw pullout performance of each lattice design in terms of ultimate pullout force (Figure 11) and withdrawal stiffness (Figure 12). The bar charts in Figure 11 reveal that the higher volume fraction (45%) generally yielded greater peak pullout forces than the lower volume fraction (30%), which is consistent with the more substantial peak loads observed in the force–displacement curves. Similarly, the withdrawal strengths of Rigid 10k lattices were increased compared to those of their Standard Grey counterparts, consistent with steeper initial slopes and more brittle-type failures of the resin material. Further detailing the trends described above in Figure 10, Figure 12 also shows screw withdrawal stiffness, in N/mm, where significant increases in this variable have been realized due to the stiffening action provided by the rigid resin and increasing volume fraction, further reflecting the much steeper initial slopes of the curves in the early loading region of Figure 10. In both figures, the various lattice topologies—Gyroid, Schwarz Diamond, Skeletal Gyroid, Skeletal Schwarz Diamond, and Skeletal Schoen I-Wrapped Package—exhibit differing degrees of strength and stiffness, underscoring the role of unit cell geometry and strut arrangement. Taken together, Figure 10, Figure 11 and Figure 12 show how the interplay among base material, relative density, and architectural design influences both the maximum load capacity of the screw–lattice interface and the stiffness response during pullout.

The screw pullout test conducted on the Standard Grey lattices identified a significant interaction between the volume fraction and unit cell design for the screw pullout strength (F(10,66) = 12.477, *p* < 0.001). Further analysis through simple main effects demonstrated that both factors had a significant impact on screw pullout strength (*p* < 0.001).

Similarly, there was a significant interaction between volume fraction and unit cell design on screw withdrawal stiffness (F(10,66) = 30.785; *p* < 0.001). Further analysis through simple main effects demonstrated that both factors had a significant impact on screw pullout strength (*p* < 0.001).

In the case of the Rigid 10k lattices, the results also highlighted a significant interaction between the volume fraction and unit cell design for the screw pullout strength (F(7,48) = 5.202; *p* < 0.001). Simple main effects analysis confirmed that both factors significantly influenced the screw pullout strength (*p* < 0.001).

There was also a significant interaction between the volume fraction and unit cell design for the screw withdrawal stiffness (F(7,48) = 25.974; *p* < 0.001). Further analysis through simple main effects demonstrated that both factors had a significant impact on the screw pullout strength (*p* < 0.001).

These analyses were then extended with Tukey’s honestly significant difference post hoc test to assess pairwise comparisons.

From the post hoc test of the screw pullout test results of the Rigid 10k material, lattices with TPMS sheet unit cell design exhibited high screw pullout strength. DS-8 was the only skeletal TPMS design that had a screw pullout strength with no significant difference from that of the TPMS sheet design (Figure 11). Considering the screw withdrawal stiffness, there was no significant difference among the lattice design for Rigid 10k lattices of 45% volume fraction (*p* = 0.05).

Pearson’s correlation coefficient revealed a strong relationship between the lattice density and screw pullout strength (Standard Grey r = 0.878; Rigid 10k r = 0.884), whereas a moderate correlation was observed between the average part thickness and compressive properties (Standard Grey r = 0.549; Rigid 10k r = 0.454), and all relationships were statistically significant (*p* < 0.01).

Similarly, a strong relationship was found between the lattice density and screw withdrawal stiffness (Standard Grey r = 0.787; Rigid 10k r =0.893), whereas a moderate correlation was observed between the average part thickness and screw withdrawal stiffness (Standard Grey r = 0.582; Rigid 10k r = 0.413), and all relationships were statistically significant (*p* < 0.01).

## 4. Discussion

### 4.1. Mechanical Responses of 3D-Printed Lattice Compared with Trabecular Bone

The elastic modulus of the mandibular trabecular bone varied between 24.9 MPa and 240.0 MPa when cortical plates were intact, whereas it ranged from 3.5 MPa to 125.6 MPa in the absence of cortical plates [39].

Most lattice designs in this study had compressive modulus values within the range of trabecular bone, except for certain groups comprising Rigid 10k material with a density of 45%.

Bone, despite being an organic substance, can often be viewed similarly to artificially engineered materials. Nevertheless, bone tends to display greater variance in measured properties compared to typical engineering materials owing to its unique synthetic process. Various factors, such as age, sex, location within the body, temperature, mineral content, water content, and diseases, including osteoporosis, can impact the properties of bone. Moreover, these variables can be affected by each other to a certain degree. For instance, the mineral content of bones can vary depending on their location in the body and the patient’s age [40].

When considering the macroscopic mechanical properties of natural bone, compact bone demonstrates the ability to endure higher stress levels (approximately 150 MPa) but shows lower strain tolerance (approximately 3%) before failure. In contrast, spongy bone can withstand lower stress levels (up to 50 MPa) but accommodates much higher strain (approximately 50%) before reaching failure [40].

From a microscopic perspective, the dominant failure mode seen in trabecular bone is characterized by the bending and buckling of the trabeculae [41].

Synthetic polyurethane blocks with varying densities are commonly used in dental implant surgical training to aid students in experiencing the tactile feel of osteotomy procedures on bones of different types [42]. Polyurethane blocks of specific densities are typically used to simulate different bone densities in the jawbone. For instance, polyurethane with densities of pcf-10 (0.16 g/cc), pcf-20 (0.32 g/cc), pcf-30 (0.48 g/cc), and pcf-40 (0.64 g/cc) are used to represent bone densities D4, D3, D2, and D1, respectively. These comparisons are based on studies that examined the mineral density at various positions in the jawbone [39,43,44]. However, these commercial models remain relatively expensive.

The application of lattice design principles allows for the customization and prediction of the structure’s mechanical properties. In addition, using 3D printing combined with functional grading lattice design allows for greater freedom in creating and designing the workpiece. Moreover, the combination with in-house hands-on models allows for the creation of various surgical scenarios.

Volume fraction constitutes the major factor affecting the mechanical properties of lattice structures. However, the feature size of the lattice structure becomes smaller as the volume fraction decreases, necessitating consideration of the resolution limitations of the 3D printer. Previous studies [13,14] have shown that specimens with smaller feature sizes tended to have greater deviations between the printed model and the CAD model in both fused deposition modeling (FDM) and SLA printing technologies.

Indeed, the two examined materials differed in the effects of the unit cell size on the mechanical response. A smaller unit cell size resulted in low compression strength in Rigid 10k structures due to lattice stiffness; thus, the reduced feature size increases the fracture probability, deforming individual struts and hence resulting in a lattice structure breakdown at lower strain values. By contrast, in the case of Standard Grey lattices, the compressive stress plateaued for structures with a smaller unit cell size because the stress was distributed, thereby causing the absorption of energy within the structure. Similar behavior can be found in trabecular bone. The failure of bone block during axial compression progresses until the cells close up sufficiently for the cell walls to touch and cause a steeply rising portion of at the end of the stress–strain curve [45]. Moreover, the Standard Grey lattices exhibited foam-like behavior, with the structure recovering up to 85–90% of its original height after compression.

Unit cell topology is another important factor affecting the mechanical properties of lattice structures. The current study leveraged this aspect to create structures with lower mechanical properties, without reducing the volume fraction, which would otherwise affect the accuracy of the printed model. Conversely, utilizing unit cell topology to design structures with higher mechanical properties at the same density can aid in reducing material usage in component manufacturing.

### 4.2. Screw Pullout Force

The pullout force exerted on an orthopedic screw is primarily dictated by the weakest structural component, which typically refers to the surrounding bone structure.

Cancellous bone is generally recognized as the weakest type of natural bone, exhibiting failure under stresses ranging from 1 to 10 MPa, whereas cortical bone typically withstands up to 100 MPa before failure, particularly when the outer layer is approximately 3.5 mm thick [46,47].

Prior investigations into bone strength have established that screw pullout strength is substantially impacted by bone density [46,48,49,50]. Comparable outcomes were noted in studies involving polyurethane foam substrates [50,51,52]. The morphology of the surrounding structure is another critical aspect influencing screw pullout strength. The pullout strength increases in bones with thicker trabeculae and a more plate-like architecture [53]. This observation is especially significant when implants have dimensions comparable to those of trabecular structures. For instance, the engagement of an orthopedic screw, where the thread depth is only slightly greater than the trabecular thickness, is significantly impacted by the bone structure surrounding the implant [54].

Pujari-Palmer et al. [55] conducted pullout tests using Sawbone samples under a setup that resembled the one used in the present study. The tests employed a 3.2 mm drill bit, a 6.5 mm screw diameter, and a 20 mm insertion depth in 40 mm tall samples. A foam material designed to replicate healthy bone with a density of 0.24 g/cm^3^ served as the control, and the highest pullout force recorded was 40 N.

Wu et al. [56] performed pullout tests on human femoral heads, reporting an average BV/TV ratio of 27.6 and pullout strength values ranging from 1134 N to 2293 N.

In another study, Wu et al. [13] used FDM-printed models based on a scaled-up model of 3D images of a human trabecular bone isolated from a femoral head. The pullout strength achieved was 1400–1900 N, which was higher than the range in the study by Grzeszczak et al. [14] (755.6 ± 146.8 N) that used the same 3D model but printed using the SLA technique, with a BV/TV ratio estimated to be 39.5%.

In this study, the pullout load recorded from 3D-printed lattices exceeded that of open-cell sawbones, which simulate osteoporotic bone density [55]. The results were equivalent to those obtained from closed-cell polyurethane foam [57]. However, the pullout load was lower than that observed for human bone with a similar volume fraction [56,58].

### 4.3. Limitations and Implications for Future Studies

In this study, the researchers conducted screw pullout tests across all groups to observe trends resulting from changes in density, unit cell topology, and unit cell size. Utilizing basic unit cell structures for design, rather than using trabecular bone model files, allows for greater design flexibility.

This offers initial insights into which structural patterns are better suited to withstand drilling and screw insertion. Additional research is required to determine the optimal candidates for hands-on models, which may involve printing structures with dense resin layers to mimic compact bone and creating models in the shape of alveolar bone to replicate anatomical structures more accurately. Moreover, further quantitative investigations may include measuring the insertion torque of screws or implants when inserted into the models.

This study could only demonstrate that photopolymer resin can be tailored to achieve mechanical properties similar to the synthetic materials used in bone replacement tests, and additional qualitative studies are required to conclude which material is suitable for use in hands-on models.

The limitations of the current study include that some of the selected designs were not scaled down to have feature thickness within the range of jawbone trabecular thickness. Future work should focus on further calibration of printing techniques to achieve thinner structures with practical production time. Second, as this study utilized only two types of photopolymer resin for comparison, the findings must be interpreted with caution and should not be broadly generalized to other 3D printers, which may yield different results. Third, the study did not conduct a direct comparison with real bone; instead, the evaluation relied on existing literature. Future research should focus on assessing the performance of lattice structures in relation to 3D models of actual trabecular bone. The investigation of mechanical response and orthopedic screw pullout in this study utilizing lattice design might motivate further studies to incorporate lattice structures into hands-on models.

## 5. Conclusions

The integration of lattice designs with AM enables the tailored production of functional structures. Recent advancements in lattice design modules, currently prevalent in most 3D-printing software, facilitate the creation of custom hands-on models meeting specific clinical case requirements. This study conducted a quantitative evaluation of 3D-printed models to explore the potential of lattice structure as an infill for dental arch models. Lattices printed from two materials differ in terms of compressive response and failure mode. Both the design and volume fraction influence the compressive modulus, screw pullout strength, and screw withdrawal stiffness of the 3D-printed lattice with interaction between both factors. Some designs with lower volume fraction give similar screw pullout strength to that of some designs with higher material usage. Screw pullout loads from all lattice designs were higher than those from open-cell polyurethane foam, and some designs gave lower values than those of human bone with a similar volume fraction. These findings demonstrate that 3D-printed lattices can be tailored to approximate different bone densities, enabling more realistic orthopedic and dental training models. Using modules that come with almost all 3D-printing software is a promising method to make custom models for medical situations without having to obtain micro-CT scans of the trabecular bone structure.

## Figures and Tables

**Table 1:** Average feature size of each lattice design.

Cell Type	Design Group	Unit Cell Size (mm)	Avg. Thickness (mm)		Volume of the CAD Model (cm^3^)	
			ρ = 30	ρ = 45	ρ = 30	ρ = 45
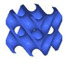 Schwarz Diamond	Dia	5.08	0.54	0.90	4.896	7.424
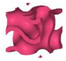 Gyroid	Gyr	5.08	0.53	0.82	4.900	7.425
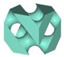 Skeletal Schwarz Diamond	DS5 DS8 DS10	5.08 3.175 2.54	1.27 0.79 0.63	1.51 0.94 0.75	4.898 4.894 4.902	7.426 7.421 7.434
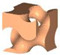 Skeletal Gyroid	GS5 GS8 GS10	5.08 3.175 2.54	1.30 0.82 0.65	1.75 1.10 0.87	4.906 4.914 4.903	7.426 7.403 7.416
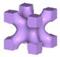 Skeletal Schoen I-Wrapped Package	I-WP5 I-WP8 I-WP10	5.08 3.175 2.54	1.64 1.02 0.82	2.10 1.32 1.05	4.837 4.851 4.837	7.429 7.452 7.420

The volume of each 3D model is based on a lattice dimension of 25.4 × 25.4 × 25.4 mm^3^. Abbreviations: Avg.—Average; CAD—computer-aided design; Dia—Schwarz Diamond; Gyr—Gyroid; DS—Skeletal Schwarz Diamond; GS—Skeletal Gyroid; I-WP—Skeletal Schoen I-Wrapped Package.

**Table 2:** Material properties as reported in Formlabs material datasheets and in-house mechanical testing.

Properties	Photopolymer Resin	
	Rigid 10k	Standard Grey
Density (g/cm^3^)	1.63	1.08
Young’s modulus (GPa)	11.6 *	3.2 *
Tensile strength (MPa)	81.7 *	31.6 *

* Values obtained from in-house mechanical testing.

**Table 3:** Screw pullout force values (N) of Standard Grey and Rigid 10k lattices.

Unit Cell Design	Standard Grey		Rigid 10k	
	ρ = 30	ρ = 45	ρ = 30	ρ = 45
Dia	312.48 ± 28.4 ^a,b,c,d *^	709.42 ± 55.2 ^f,g^	1334.57 ± 120.5 ^c,d^	2706.90 ± 133.9 ^j^
Gyr	257.62 ± 15.1 ^a,b,c^	752.13 ± 33.7 ^f,g,h^	1087.80 ± 118.3 ^c^	2494.08 ± 196.4 ^i,j^
DS-5	382.98 ± 20.4 ^c,d,e^	913.80 ± 92.3 ^j,k,l^	N/A	N/A
GS-5	404.23 ± 69.2 ^d,e^	721.15 ± 80.8 ^f,g,h^	N/A	N/A
I-WP-5	458.59 ± 47.0 ^e^	959.35 ± 87.0 ^k,l^	N/A	N/A
DS-8	380.12 ± 53.9 ^c,d,e^	854.63 ± 41.0 ^i,j,k^	980.27 ± 116.2 ^c^	2337.70 ± 215.1 ^h,i,j^
GS-8	255.09 ± 33.4 ^a,b,c^	996.24 ± 67.1 ^l^	236.22 ± 73.0 ^a,b^	1978.13 ± 225.7 ^e,f,g^
I-WP-8	369.14 ± 36.2 ^b,c,d,e^	800.68 ± 55.3 ^g,h,i^	507.63 ± 93.0 ^b^	2295.74 ± 255.0 ^g,h^
DS-10	215.44 ± 31.6 ^a^	703.53 ± 50.8 ^f,g^	222.56 ± 48.6 ^a,b^	1692.97 ± 142.6 ^d,e^
GS-10	241.86 ± 15.4 ^a,b^	470.22 ± 27.6 ^e^	92.33 ± 30.0 ^a^	2089.84 ± 187.0 ^f,g^
I-WP-10	224.17 ± 37.3 ^a^	659.15 ± 56.8 ^f^	358.60 ± 44.6 ^a,b^	1708.97 ± 203.9 ^d,e,f^

Data are presented as the mean ± SD. * Similar statistical letters within a parameter (column) indicate non-significant difference among the lattice design of each material in Tukey’s HSD post hoc test. Abbreviations: Gyr—Gyroid; Dia—Schwarz Diamond; GS—Skeletal Gyroid; DS—Skeletal Schwarz Diamond; I-WP—Skeletal Schoen I-Wrapped Package; N/A—not available.

**Table 4:** Withdrawal stiffness values (N/mm) of Standard Grey and Rigid 10k lattices.

**Unit Cell Design**	**Standard Grey**		**Rigid 10k**	
	**ρ = 30**	**ρ = 45**	**ρ = 30**	**ρ = 45**
Dia	362.15 ± 48.4 ^a *^	762.55 ± 37.0 ^c,d^	2378.63 ± 177.2 ^d^	3404.48 ± 250.8 ^e,f^
Gyr	290.53 ± 58.5 ^a^	923.89 ± 53.4 ^e,f^	2123.88 ± 57.5 ^d^	3619.18 ± 187.6 ^f^
DS-5	605.83 ± 44.9 ^b^	1233.40 ± 91.1 ^g^	N/A	N/A
GS-5	657.62 ± 50.2 ^b,c^	1026.53 ± 42.2 ^f^	N/A	N/A
I-WP-5	538.73 ± 67.56 ^b^	1209.59 ± 34.5 ^g^	N/A	N/A
DS-8	641.46 ± 40.0 ^b,c^	1293.08 ± 35.8 ^g,h^	2002.83 ± 209.2 ^d^	3391.50 ± 213.1 ^e,f^
GS-8	353.41 ± 69.1 ^a^	1384.13 ± 77.5 ^h^	983.58 ± 165.4 ^b,c^	3368.95 ± 208.8 ^e,f^
I-WP-8	630.40 ± 59.1 ^b,c^	1301.30 ± 74.5 ^g,h^	1188.82 ± 195.5 ^c^	3306.08 ± 197.3 ^e,f^
DS-10	267.63 ± 24.8 ^a^	758.81 ± 67.5 ^c,d^	1169.59 ± 180.6 ^c^	3243.85 ± 161.4 ^e,f^
GS-10	303.58 ± 23.6 ^a^	524.18 ± 39.2 ^b^	547.57 ± 30.9 ^a^	3398.13 ± 172.6 ^e,f^
I-WP-10	313.21 ± 12.8 ^a^	826.42 ± 58.7 ^d,e^	644.57 ± 47.6 ^a,b^	3066.78 ± 57.2 ^e^

Data are presented as the mean ± SD. * Similar statistical letters within a parameter (column) indicate non-significant difference among the lattice design of each material in Tukey’s HSD post hoc test. Abbreviations: Gyr—Gyroid; Dia—Schwarz Diamond; GS—Skeletal Gyroid; DS—Skeletal Schwarz Diamond; I-WP—Skeletal Schoen I-Wrapped Package.

## Data Availability

The datasets supporting the conclusions of this article are included within the article. The raw data are available upon reasonable request from the corresponding author.

## Abbreviations

The following abbreviations are used in this manuscript:

AM	Additive manufacturing
BV/TV	Bone tissue volume to total volume
CAD	Computer-aided design
CBCT	Cone beam computed tomography
CT	Computed tomography
F-d	Force–displacement
FDM	Fused deposition modeling
SLA	Stereolithography (printing technology)
STL	Stereolithography (file type)
TPMS	Triply periodic minimal surfaces
